# Supplementary material for: Photoprocesses in Derivatives of 1,4- and 1,3-Diazadistyryldibenzenes
Source: Int J Mol Sci. 2022 Dec 5;23(23):15346. doi: 10.3390/ijms232315346 (PMC9736992; doi:10.3390/ijms232315346)
Supplement: Supplementary file 1 [file ijms-23-15346-s001.zip › ijms-1979611-supplementary.pdf]

Supplementary Materials

NMR spectra of compounds *1* and *2* 2

Natural orbitals singly occupied in the S_1 and its closest triplet state 3

Energy diagrams for *1* and *2* calculated by BHHLYP 3

Atomic coordinates of all structures in study 4

Trans-1, ground state 4

Trans-1, excited state 5

Cis-1, ground state 6

Cis-1, excited state 7

DHP-*1*, ground state 8

DHP-*1*, excited state (near-CI structure) 9

Phen-*1*, ground state 10

Trans-to-cis-1 TS, excited state 11

Cis-to-trans-1 TS, excited state 12

Trans-to-cis-1 near-CI region 13

Trans-2, ground state 14

Trans-2, excited state 15

Cis-2, ground state 16

Cis-2, excited state 17

DHP-*2*, ground state 18

DHP-*2*, excited state (near-CI structure) 19

Phen-*2*, ground state 20

Trans-to-cis-2 TS, excited state 21

Cis-to-trans-2 TS, excited state 22

Trans-to-cis-2 near-CI region 23

NMR spectra of compounds *1* and *2*

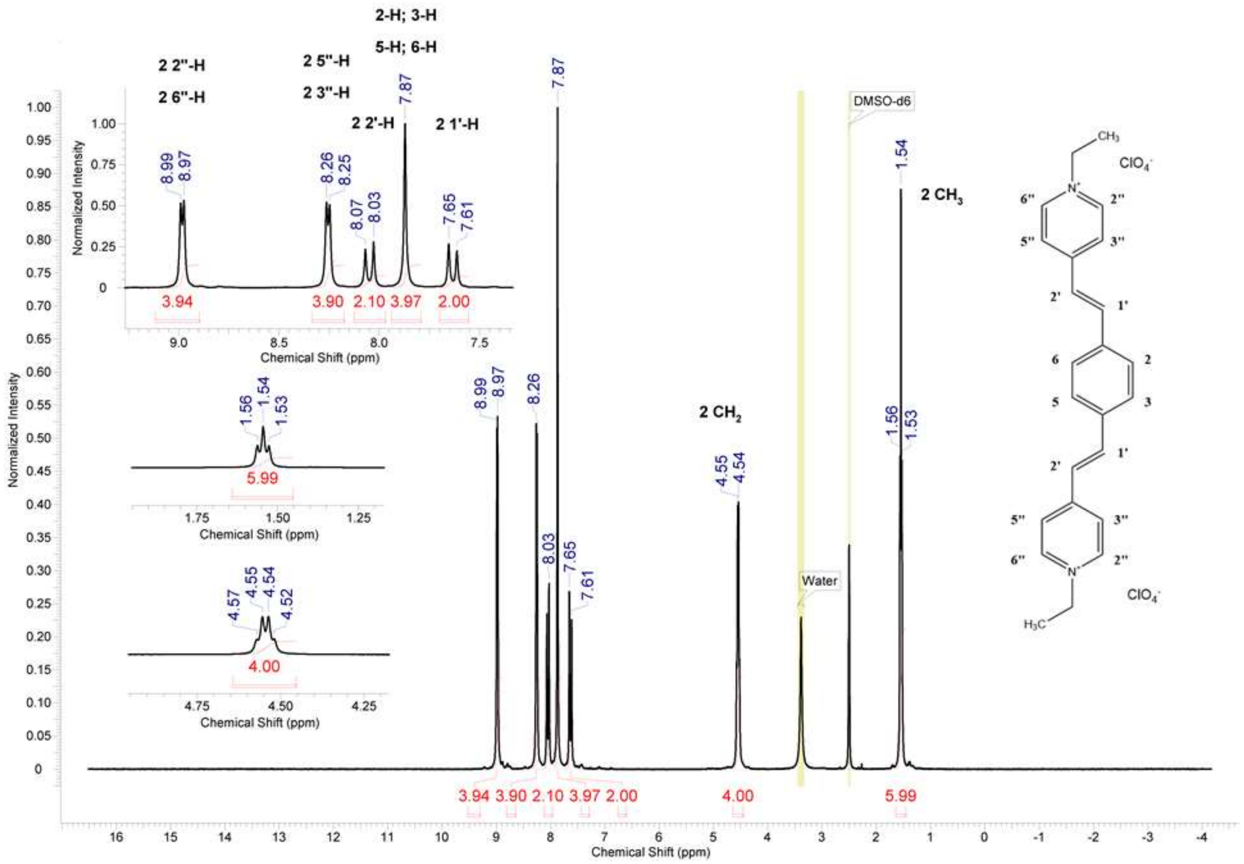

Figure S1. ^{1}H NMR spectrum of ***1***.

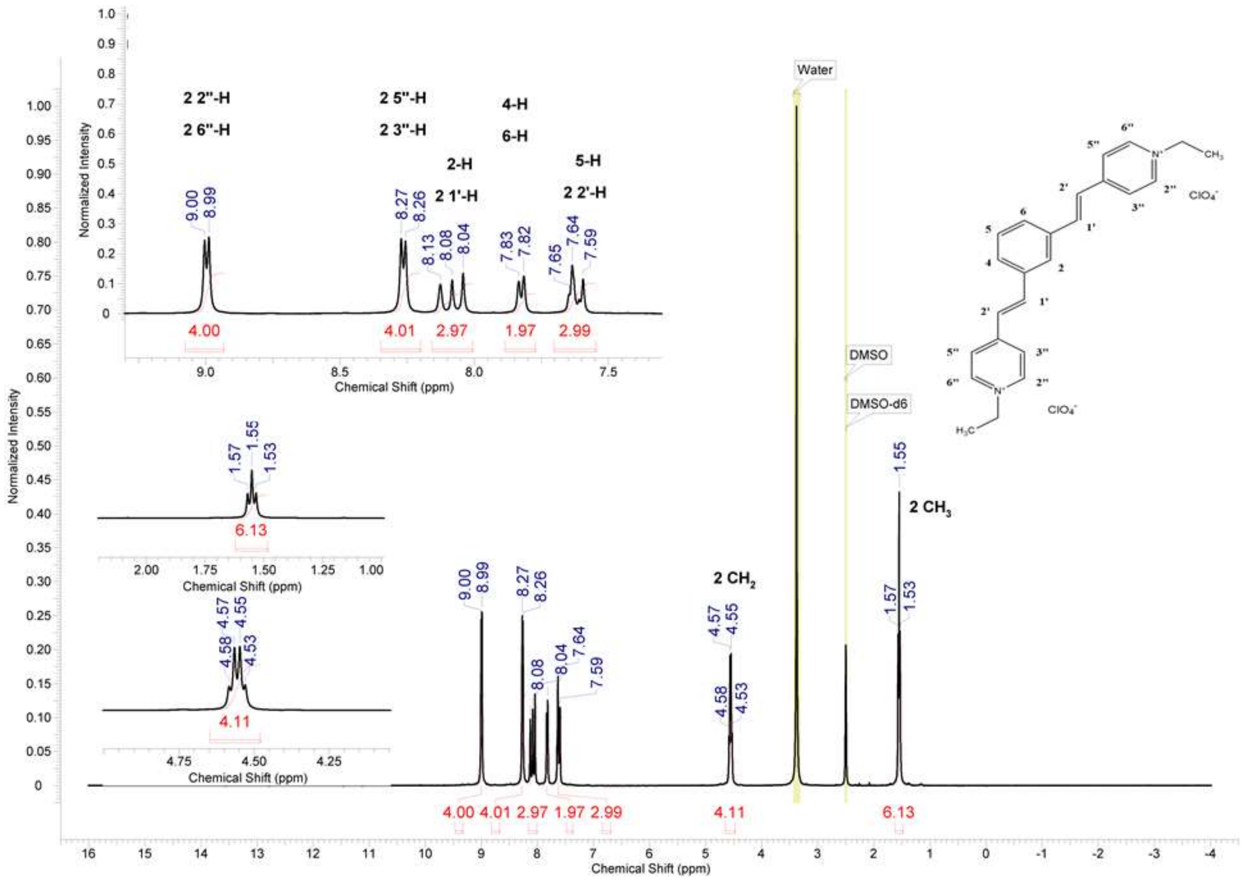

Figure S2. ^{1}H NMR spectrum of ***2***.

Natural orbitals singly occupied in the S_1 and its closest triplet state

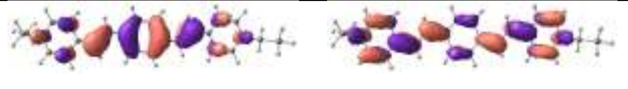	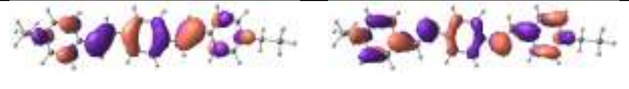
(*Trans*-***1***)*, S_1	(*Trans*-***1***)*, T_2
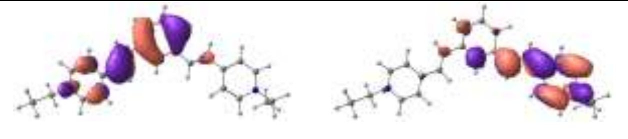	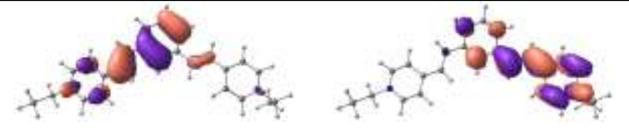
(*Trans*-***2***)*, S_1	(*Trans*-***2***)*, T_3

Figure S3. Natural orbitals singly occupied in the S_1 and its closest triplet state in ***1*** and ***2***.

Energy diagrams for *1* and *2* calculated by BHHLYP

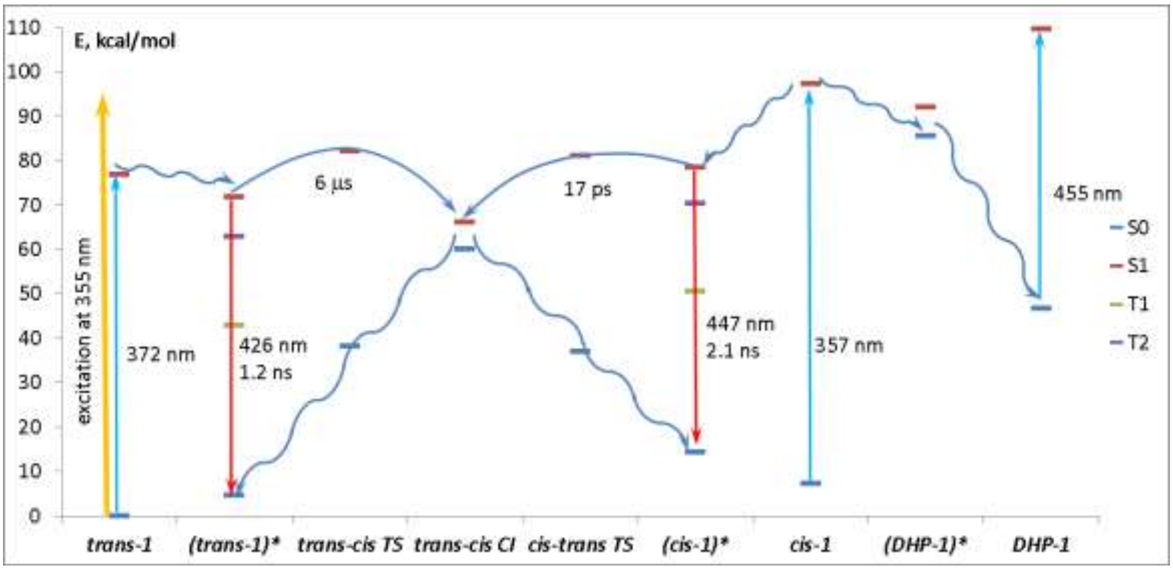

a

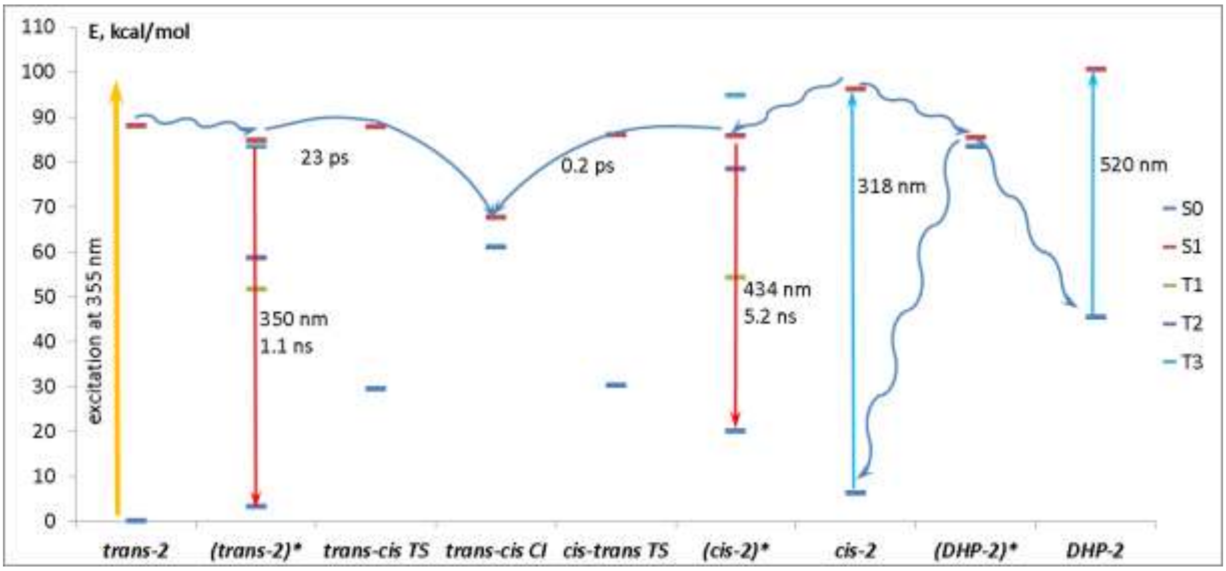

b

Figure S4. Energy diagrams for ***1***(a) and ***2***(b) calculated by BHHLYP

Atomic coordinates of all structures in study

*Trans-**1***, ground state

C	0.937710924	3.516864053	1.450899390
C	0.528771476	3.495510477	0.100792682
C	0.912259423	2.278579783	2.122611086
C	0.133115071	2.324977931	-0.497614343
C	0.505760386	1.135465186	1.475112481
H	0.522890774	4.405803438	-0.489021505
H	1.208506977	2.187901181	3.160681688
H	-0.185084091	2.281372319	-1.533371888
C	1.347750615	4.771149360	2.046002755
H	1.298020317	5.630530190	1.382550298
C	1.771121596	4.933412284	3.320772231
H	1.818896965	4.063404600	3.973737695
C	2.186874991	6.175449372	3.950074281
C	2.600278851	6.135894132	5.295688317
C	2.197785022	7.416306549	3.289012015
C	3.007493500	7.279054475	5.956984981
C	2.604311285	8.559543441	3.950507384
C	3.018409410	8.519886546	5.295898222
H	2.598649412	5.186961608	5.824987275
H	1.886844702	7.493990838	2.252481194
H	3.316641816	7.201838406	6.994093131
H	2.604758056	9.508710475	3.421629559
C	3.432310989	9.762447483	5.925364437
H	3.370405517	10.634524639	5.276360994
C	3.871189513	9.923136656	7.195042691
H	3.941543441	9.060924693	7.852941065
C	4.274606237	11.179537833	7.790574244
C	4.260891155	12.423276671	7.129986693
C	4.718610664	11.196338483	9.130208844
C	4.663243071	13.568762710	7.777364003
C	5.109181412	12.368163317	9.727993935
H	3.936023149	12.517785527	6.100884282
H	4.759143632	10.280638300	9.710214304
H	4.658723267	14.532693499	7.286730803
H	5.454328967	12.410087066	10.755365687
N	5.081965096	13.543968800	9.059823331
N	0.121440675	1.155442779	0.180927786
C	5.521085578	14.767924111	9.789176075
H	4.878832237	14.842713772	10.671699057
H	6.541751896	14.569486929	10.129472233
C	5.469327577	16.047565078	8.986117014
H	4.454611799	16.304125776	8.668548211
H	6.134685831	16.027056900	8.118235538
H	5.814972907	16.856778291	9.634207362
H	0.480606475	0.175366944	1.972928039
C	-0.365065223	-0.064186333	-0.523274581
H	-1.454469064	0.024256718	-0.589026666
H	0.039180723	-0.003656660	-1.536753532
C	0.034114508	-1.373268804	0.121971219
H	-0.456861896	-1.543017922	1.083977594

H	1.117926884	-1.461225849	0.241236704
H	-0.288362875	-2.180692337	-0.540152772

Trans-***1***, excited state

C	0.944794718	3.535614955	1.457985805
C	0.522672479	3.528568871	0.106045818
C	0.938561800	2.280792809	2.121203767
C	0.135434822	2.363696893	-0.506204531
C	0.541545518	1.147240448	1.460740096
H	0.503446194	4.447382616	-0.470796435
H	1.247490564	2.181145375	3.154944685
H	-0.190123017	2.326798186	-1.539603789
C	1.348696389	4.770159871	2.067991431
H	1.294008471	5.642109288	1.420984096
C	1.786195163	4.928071031	3.364061222
H	1.841806837	4.055239680	4.010412868
C	2.188197838	6.157909359	3.965910431
C	2.617782046	6.147112285	5.324620488
C	2.186857882	7.413262677	3.282118803
C	3.015892405	7.292101723	5.960609882
C	2.585156226	8.558224871	3.918056042
C	3.013932138	8.547560394	5.277023474
H	2.627381748	5.203508578	5.862523798
H	1.868339925	7.470544434	2.247123498
H	3.335759021	7.234570814	6.995175504
H	2.576379508	9.501688312	3.379890031
C	3.415144806	9.777489009	5.879238107
H	3.363684245	10.649701180	5.231707116
C	3.846204419	9.936213362	7.177356378
H	3.894348411	9.065206051	7.826154569
C	4.250243055	11.170524084	7.787739192
C	4.269228897	12.423400920	7.121032052
C	4.659671184	11.179313049	9.143536544
C	4.666022919	13.556729202	7.781931551
C	5.047069274	12.343984541	9.756169365
H	3.971702594	12.521391091	6.083792015
H	4.669541231	10.261974837	9.722957973
H	4.690484573	14.529224719	7.303066169
H	5.362813728	12.382145537	10.792558911
N	5.046832576	13.523858845	9.084132026
N	0.147879320	1.181929951	0.162476347
C	5.526736061	14.752377887	9.746385878
H	4.971477885	15.589031873	9.314763263
H	5.242835714	14.682236696	10.799572324
C	7.027419228	14.935822198	9.584061873
H	7.308184417	15.020789585	8.530569797
H	7.582113956	14.104384781	10.027776871
H	7.333208581	15.854754405	10.090873035
H	0.527098989	0.173227314	1.936883948
C	-0.331584411	-0.046851350	-0.499694572
H	-0.047278251	0.022884823	-1.552841482
H	0.223780351	-0.883045071	-0.067419544

C	-1.832183604	-0.230864156	-0.337362687
H	-2.387319652	0.599622632	-0.782362618
H	-2.113075659	-0.314536704	0.716170989
H	-2.137471636	-1.150344749	-0.843486609

Cis-***1***, ground state

C	-1.337581583	14.927842085	1.113786429
C	-1.274810043	15.065726110	-0.285717133
C	-2.300382700	15.705719883	1.788009195
C	-2.125285096	15.923897087	-0.941135256
C	-3.120973852	16.550963305	1.082860655
H	-0.553036759	14.501768948	-0.861753143
H	-2.408984909	15.662361740	2.863179550
H	-2.110315917	16.060880319	-2.013211652
C	-0.421255819	14.020923370	1.774135601
H	0.269544570	13.504748361	1.113648889
C	-0.383787186	13.799868323	3.106954356
H	-1.089705059	14.320726735	3.749602077
C	0.531689033	12.911556414	3.805774140
C	0.418749170	12.795926205	5.203035977
C	1.513420383	12.145226159	3.150246038
C	1.260169702	11.962873941	5.924093415
C	2.347576239	11.307435234	3.869710096
C	2.256558180	11.218130894	5.272218855
H	-0.350387469	13.361983992	5.718845390
H	1.621334095	12.202256764	2.072563981
H	1.128412251	11.868290673	6.996714360
H	3.094001166	10.715181055	3.349348863
C	3.144956196	10.283461575	5.963737302
H	3.397938888	9.404458015	5.373440897
C	3.674512008	10.306618889	7.207528782
H	4.211097163	9.412023095	7.513327971
C	3.684273906	11.355573465	8.220107982
C	3.584706433	12.732871156	7.947759952
C	3.898358230	10.987391778	9.561235584
C	3.672618436	13.646848099	8.970712355
C	3.969893760	11.944207567	10.547032944
H	3.448373931	13.099285707	6.939431355
H	4.000555776	9.944459922	9.833072251
H	3.606057834	14.713181962	8.809374672
H	4.120534550	11.702302100	11.589846865
N	3.853223371	13.251605423	10.247362756
N	-3.029735667	16.655076186	-0.259903735
C	4.002919388	14.263952068	11.310245674
H	3.379198658	15.110436014	11.026130731
H	3.590913717	13.829327623	12.220510299
C	5.454073079	14.667193909	11.479840508
H	5.851684550	15.102873609	10.560710855
H	6.072279291	13.810555850	11.756122869
H	5.527318864	15.413269321	12.273046398
H	-3.867032714	17.173080760	1.556086620
C	-3.966911143	17.527779354	-0.992219909

H	-4.175523347	18.377458617	-0.342056308
H	-3.434352960	17.893440101	-1.869668053
C	-5.232454781	16.783742092	-1.369855873
H	-5.755192107	16.421994737	-0.482203664
H	-5.010299938	15.932156253	-2.016230391
H	-5.898019878	17.460385010	-1.908427657

Cis-***1***, excited state

C	-1.357578347	14.951493365	1.080905250
C	-1.361244041	14.982651322	-0.333665740
C	-2.294922720	15.795843777	1.726658179
C	-2.231862890	15.792457228	-1.019372219
C	-3.142236255	16.584963410	0.991446105
H	-0.671130929	14.367407500	-0.901457281
H	-2.368876809	15.844612499	2.806510774
H	-2.256939897	15.838042950	-2.102285178
C	-0.433022397	14.090310897	1.766011932
H	0.210397672	13.501444801	1.117382556
C	-0.308747481	13.956991060	3.126826016
H	-0.947984405	14.552139813	3.775266073
C	0.596340494	13.080329846	3.802745355
C	0.587621549	13.049073462	5.223462524
C	1.494493132	12.198861813	3.124598813
C	1.417684428	12.215990221	5.929735304
C	2.312127005	11.354097368	3.824801393
C	2.327854693	11.342177948	5.250966653
H	-0.113881696	13.684538146	5.756831721
H	1.529990161	12.183247839	2.040882508
H	1.343011082	12.174165598	7.010725300
H	2.979069061	10.683651963	3.290572792
C	3.162510992	10.394559590	5.912015625
H	3.496809451	9.571598652	5.280804710
C	3.554631268	10.302200974	7.237167650
H	3.928381943	9.324919767	7.540795685
C	3.632753354	11.309223854	8.257087748
C	3.713046177	12.704661929	7.994535863
C	3.728400657	10.921105065	9.617564887
C	3.841470897	13.598020911	9.027822955
C	3.844198321	11.858458805	10.610321403
H	3.728256706	13.082235029	6.978969520
H	3.697794116	9.872289191	9.894418614
H	3.925974128	14.666737713	8.864416601
H	3.907604978	11.594548849	11.659757862
N	3.889050804	13.188796914	10.320075001
N	-3.111862544	16.588529517	-0.363923282
C	4.100511320	14.168496050	11.402102796
H	3.608328500	15.096249004	11.098969340
H	3.572374558	13.793754037	12.282861058
C	5.577218682	14.388429418	11.691087894
H	6.102208471	14.778711960	10.814744260
H	6.064874316	13.461816964	12.006290867
H	5.680996389	15.116017013	12.500220332

H	-3.870671945	17.242002495	1.453354307
C	-4.082071154	17.403535934	-1.124418615
H	-4.267882158	18.309007486	-0.541292157
H	-3.588224219	17.708282248	-2.050455671
C	-5.367845468	16.641419273	-1.400883309
H	-5.868112786	16.349301135	-0.473386090
H	-5.180892657	15.743591142	-1.996509788
H	-6.049764326	17.283642183	-1.964183403

DHP-***1***, ground state

C	-1.469752108	14.814176384	1.050748356
C	-1.417771043	14.837091538	-0.355000244
C	-2.283552576	15.779666220	1.674542510
C	-2.124764537	15.780339326	-1.063430687
C	-2.969796169	16.696458350	0.917563841
H	-0.808276429	14.122963688	-0.893369133
H	-2.387451564	15.819786984	2.749604004
H	-2.101485803	15.846589867	-2.141928319
C	-0.691888562	13.826985761	1.775579899
H	-0.206577831	13.075362341	1.160173815
C	-0.524575568	13.825108748	3.116122031
H	-0.992173571	14.607175025	3.709538388
C	0.269515763	12.873515398	3.877139922
C	0.439545176	13.077746589	5.207840382
C	0.921348238	11.731437573	3.243588363
C	1.194338090	12.119481814	6.072290752
C	1.810661467	10.963076466	3.918003129
C	2.126303536	11.214442338	5.297477934
H	-0.043789734	13.927853353	5.680611426
H	0.723829527	11.525829307	2.197524511
H	0.433038623	11.440891604	6.517068040
H	2.345258840	10.167870165	3.406943211
C	3.165782281	10.597207991	5.934809023
H	3.760399247	9.862948345	5.400396674
C	3.477585477	10.882902325	7.298595058
H	4.252060903	10.308577615	7.798437182
C	2.862954767	11.898295952	7.972040398
C	1.905228669	12.812088559	7.234919045
C	3.174581563	12.257407531	9.317870787
C	0.922822348	13.407273241	8.178296943
C	2.418135748	13.154322503	9.991220974
H	2.495230128	13.663535743	6.832173736
H	4.005543010	11.782620622	9.827624515
H	-0.060175553	13.723140494	7.849885186
H	2.593098565	13.471604160	11.007456319
N	1.221044020	13.623106252	9.418424633
N	-2.884264996	16.694725507	-0.429250246
C	0.276691980	14.324045600	10.312302633
H	-0.648697550	14.442332607	9.751546384
H	0.087052585	13.657270236	11.155005832
C	0.832863207	15.659280416	10.762504956
H	1.035021262	16.314190540	9.913254194

H	1.749910633	15.543049300	11.343865170
H	0.093472320	16.143537570	11.402560463
H	-3.598814945	17.460934212	1.349875618
C	-3.675357548	17.664080962	-1.211114857
H	-3.736999836	18.572746291	-0.612854500
H	-3.101325916	17.886939316	-2.110100658
C	-5.050624597	17.115089658	-1.538790600
H	-5.609084512	16.886224537	-0.628962969
H	-4.977433511	16.208026902	-2.142107180
H	-5.609296076	17.861033726	-2.106395793

DHP-***1***, excited state (near-CI structure)

C	-1.402687348	14.987896344	1.075080508
C	-1.204428316	15.207073622	-0.301855650
C	-2.509843956	15.635955233	1.658057199
C	-2.051988061	16.023560604	-1.015188228
C	-3.325870169	16.440366352	0.897938483
H	-0.374674386	14.743675029	-0.824792232
H	-2.755116295	15.517316193	2.706684598
H	-1.920255331	16.214344587	-2.074502517
C	-0.485255571	14.120204537	1.791190249
H	0.251002464	13.624091850	1.164837202
C	-0.474189969	13.906423385	3.125502289
H	-1.191583877	14.429841305	3.754986616
C	0.434950464	13.019641996	3.843451380
C	0.370031287	12.980954105	5.225379272
C	1.368449004	12.175497456	3.168803975
C	1.175414839	12.083581259	6.027308889
C	2.173379638	11.306508775	3.871564712
C	2.107757711	11.219633916	5.274645494
H	-0.355707103	13.612726054	5.732998567
H	1.453482418	12.195645713	2.088292693
H	0.552878562	11.469470878	6.693037441
H	2.888790207	10.688089444	3.336380699
C	2.945492011	10.371614048	6.017357151
H	3.533190397	9.620577104	5.494365129
C	3.035462245	10.476796758	7.409917721
H	3.491799708	9.674607133	7.982456093
C	2.612975005	11.624295727	8.079471532
C	2.156776440	12.811278453	7.289734815
C	2.575526158	11.731880027	9.498238382
C	1.541377725	13.863618721	8.047416413
C	1.867222921	12.734514851	10.090594437
H	2.975553149	13.196956667	6.671261758
H	3.013699253	10.956881721	10.116588506
H	1.261925004	14.801659077	7.583560383
H	1.721176175	12.771326719	11.164553849
N	1.274407545	13.745484732	9.372995106
N	-3.096195516	16.634385841	-0.418867042
C	0.529092424	14.788138375	10.094275743
H	-0.021115914	15.364954213	9.347360874
H	-0.212644038	14.291036906	10.727322883

C	1.428416734	15.698567420	10.916057590
H	2.161058990	16.208043106	10.283982068
H	1.966658935	15.150781205	11.694594932
H	0.818798803	16.458362445	11.411704465
H	-4.185434557	16.952383847	1.315901063
C	-4.028274571	17.464626586	-1.220866364
H	-4.393568340	18.256847540	-0.563190149
H	-3.433353544	17.940396538	-2.004043585
C	-5.163359500	16.638672612	-1.798403419
H	-5.764273153	16.172429195	-1.012680965
H	-4.793184341	15.857871247	-2.468507043
H	-5.819374952	17.293690424	-2.377389011

Phen-***1***, ground state

C	-1.409776958	14.889862996	1.044528815
C	-1.355124981	14.961368388	-0.358730535
C	-2.326026925	15.738890414	1.693323810
C	-2.162295681	15.840062193	-1.042428216
C	-3.111037846	16.594286358	0.960164456
H	-0.669251968	14.336361114	-0.915042143
H	-2.434052617	15.735553734	2.768502618
H	-2.143807582	15.941671920	-2.117951269
C	-0.529537491	13.970242555	1.740432123
H	0.051935781	13.314696001	1.099432562
C	-0.384617464	13.913088463	3.081455909
H	-0.949797021	14.599305317	3.707741629
C	0.502901571	13.013334640	3.804599764
C	0.641395459	13.184942370	5.179665378
C	1.229742458	11.976842279	3.166106677
C	1.491093280	12.364204387	5.939896707
C	2.061110889	11.158543574	3.891360291
C	2.214581789	11.331132283	5.286370403
H	0.082866685	13.982824311	5.656710211
H	1.125605030	11.821285867	2.098073357
H	2.606285480	10.360852682	3.397824760
C	3.083262819	10.480126622	6.038589866
H	3.613585102	9.694411015	5.509668713
C	3.254028475	10.634103716	7.382726034
H	3.915787472	9.980209523	7.939352196
C	2.552098856	11.659960339	8.078956327
C	1.669638286	12.521257370	7.369001366
C	2.701019518	11.851835215	9.469915322
C	0.991943273	13.500190157	8.095708004
C	2.004520812	12.838379820	10.108521232
H	3.365872048	11.214819200	10.039683024
H	0.300306463	14.191834901	7.636836209
H	2.077724985	13.033761191	11.168738841
N	1.158165360	13.643143620	9.410464301
N	-3.022005328	16.641949595	-0.385253552
C	0.430107671	14.707132705	10.133583025
H	-0.346687102	15.060914536	9.457845017
H	-0.055857187	14.231720453	10.986460954

C	1.356849528	15.825208016	10.564230068
H	1.829532375	16.303254965	9.704668500
H	2.136100211	15.459406999	11.236446825
H	0.774128301	16.576448703	11.099962703
H	-3.826836861	17.265350208	1.412322716
C	-3.915456430	17.536926692	-1.145504915
H	-4.079131344	18.417530256	-0.525254817
H	-3.366214072	17.845298978	-2.034639468
C	-5.216749829	16.842927991	-1.496556114
H	-5.753597640	16.535002988	-0.597376158
H	-5.035846139	15.961584454	-2.114862432
H	-5.850772907	17.531960579	-2.056620145

*Trans-to-cis-**1*** TS, excited state

C	7.012005997	8.117281225	-1.091575813
C	6.458877219	7.929157447	-2.377656125
C	8.422516316	8.070178094	-1.007021678
C	7.265268498	7.713328040	-3.468199396
C	9.180815609	7.851518232	-2.129853327
H	5.384261696	7.948054943	-2.524771703
H	8.941943255	8.200791750	-0.065329356
H	6.868907322	7.564305873	-4.466390837
C	6.132263368	8.341638505	0.029074918
H	5.074040607	8.356050040	-0.218773634
C	6.518594963	8.531656835	1.321350138
H	7.579454928	8.517639582	1.562642274
C	5.647897668	8.755101823	2.446420962
C	6.235749853	8.932997758	3.726283880
C	4.231351531	8.810322039	2.340591479
C	5.468603913	9.174878610	4.836358527
C	3.454241203	9.043127274	3.442837661
C	4.043921700	9.242768365	4.731600952
H	7.317015585	8.884665253	3.819849243
H	3.749179597	8.669050792	1.379584425
H	5.930562270	9.326286788	5.808465829
H	2.372885317	9.089498074	3.346594362
C	3.239789814	9.527521515	5.834418496
H	2.158166111	9.555234038	5.656088180
C	3.703256379	9.860237538	7.158177531
H	4.260400467	10.792779554	7.273779962
C	3.389371753	9.144433967	8.317043335
C	2.642585214	7.924324160	8.299092230
C	3.777283174	9.615611844	9.610325847
C	2.320672279	7.288681987	9.465923125
C	3.420040036	8.932151595	10.737158618
H	2.313323951	7.491906330	7.359825412
H	4.346041203	10.533665647	9.713407104
H	1.750867668	6.366131165	9.484978164
H	3.689781223	9.270473530	11.731461934
N	2.691067499	7.783932113	10.674875205
N	8.611767637	7.671790609	-3.345084016
C	2.361494992	7.052611822	11.911551411

H	2.177774522	7.798836086	12.689077485
H	1.418277350	6.529359061	11.735611571
C	3.465216818	6.088139985	12.316931938
H	4.405665915	6.613417303	12.505766758
H	3.636475022	5.330633165	11.546925085
H	3.174667215	5.575341883	13.237574997
H	10.263773262	7.808833600	-2.099015158
C	9.459584852	7.497442774	-4.545182075
H	10.369034752	6.985251076	-4.222143955
H	8.925771468	6.821683055	-5.217971253
C	9.770798966	8.825371000	-5.214380820
H	10.317038419	9.494290252	-4.543585532
H	8.859373780	9.330149058	-5.546514640
H	10.395116547	8.645123847	-6.093183193

*Cis-to-trans-**1*** TS, excited state

C	6.986522680	8.144154866	-0.991070765
C	6.450570870	7.849376693	-2.264044255
C	8.394903532	8.091410814	-0.880380500
C	7.270708165	7.527280059	-3.317903141
C	9.167195006	7.763836181	-1.966678356
H	5.378579089	7.865281976	-2.429337464
H	8.901416575	8.297026409	0.054934852
H	6.887388460	7.292565283	-4.304671598
C	6.091677673	8.463198981	0.094468040
H	5.036721844	8.449541048	-0.166676222
C	6.459305449	8.756816272	1.372689779
H	7.516862156	8.770425463	1.628401499
C	5.569403550	9.040820624	2.468764576
C	6.133336728	9.289021846	3.745868970
C	4.152258165	9.060335134	2.341066677
C	5.344855128	9.536575713	4.841779542
C	3.353556939	9.299242329	3.425752824
C	3.917427881	9.544024086	4.718539569
H	7.213926371	9.265891648	3.856622514
H	3.687356464	8.873759551	1.379338881
H	5.790703321	9.699452834	5.818589353
H	2.272589841	9.302188529	3.316171965
C	3.081835762	9.771056984	5.811897376
H	2.005056261	9.680869720	5.632732611
C	3.486818155	10.029492876	7.166864355
H	3.147764798	9.309519022	7.915452564
C	4.175570432	11.154875165	7.623723960
C	4.594752353	12.224914304	6.766803581
C	4.504449800	11.303359022	9.009137459
C	5.262282227	13.302905046	7.281529502
C	5.177450725	12.403766940	9.452827290
H	4.371768443	12.205035750	5.705949850
H	4.221657876	10.538141661	9.724291193
H	5.578298505	14.135947187	6.663019644
H	5.437730314	12.544805437	10.495949361
N	5.561448931	13.397688311	8.601407006

N	8.614181142	7.480864149	-3.169475799
C	6.215090125	14.606014735	9.135123735
H	6.889136389	14.284035462	9.933671968
H	6.833479581	15.019469484	8.334434860
C	5.204633600	15.625329261	9.638928547
H	4.595429383	15.217519059	10.450349440
H	4.539981193	15.957363797	8.836516814
H	5.736293197	16.499583149	10.023629454
H	10.248848470	7.709683459	-1.914184027
C	9.479500493	7.181006017	-4.331934414
H	10.359143904	6.658070522	-3.948976882
H	8.931843355	6.478041293	-4.964316196
C	9.861050609	8.439855192	-5.092102032
H	10.417888941	9.137101283	-4.459858450
H	8.979585450	8.952499443	-5.487230394
H	10.499073845	8.167640181	-5.936703892

*Trans-to-cis-**1*** near-CI region

C	7.024435661	8.109630047	-1.109288233
C	6.467009147	7.885611344	-2.385197175
C	8.433894761	8.116626076	-1.037008825
C	7.272095182	7.686726507	-3.481814898
C	9.190189612	7.912682713	-2.165685660
H	5.391079744	7.862455457	-2.521900613
H	8.956439059	8.278195827	-0.101881594
H	6.872882006	7.510791816	-4.474589888
C	6.143022503	8.314580925	0.018673672
H	5.083109539	8.286734302	-0.219844588
C	6.532178346	8.534094416	1.300872659
H	7.595096693	8.561509614	1.532451635
C	5.662255816	8.743902459	2.433489195
C	6.257578671	8.958986231	3.701145690
C	4.244481423	8.748240405	2.338495491
C	5.492931935	9.187517742	4.819481433
C	3.470474794	8.970342046	3.444998525
C	4.068293281	9.207318889	4.724380830
H	7.340917571	8.947398904	3.783022837
H	3.758895941	8.577623404	1.383864083
H	5.953450254	9.361144468	5.788073679
H	2.387185548	8.977887471	3.360585066
C	3.277975785	9.473356894	5.837126976
H	2.191902215	9.460806988	5.679607871
C	3.779025419	9.835764916	7.149648709
H	3.915377883	10.898570365	7.362216458
C	3.981396340	8.934220202	8.182416798
C	3.811935961	7.519430769	8.018781773
C	4.361223500	9.364193629	9.497348259
C	3.984422116	6.672269749	9.076701797
C	4.516614762	8.462941784	10.505769827
H	3.537918808	7.104225870	7.054910931
H	4.513823924	10.416965237	9.708452844
H	3.855928595	5.599316092	8.985682106

H	4.791342710	8.760405991	11.511564952
N	4.322732938	7.125755097	10.310957921
N	8.617300659	7.697413061	-3.370795796
C	4.542507566	6.180665446	11.419108967
H	4.206606660	6.675348009	12.334402007
H	3.880983768	5.327269575	11.249629610
C	5.994542367	5.743115256	11.527483542
H	6.655550754	6.594971385	11.710271442
H	6.325249367	5.232193169	10.618805499
H	6.102481311	5.048059163	12.364343203
H	10.274282408	7.911024143	-2.145354864
C	9.460885802	7.540239288	-4.578057478
H	10.391793945	7.066472669	-4.257881818
H	8.946892926	6.836812558	-5.237592766
C	9.714786250	8.871458305	-5.264545977
H	10.241445069	9.568504650	-4.606838632
H	8.781944771	9.337638522	-5.593667213
H	10.337282010	8.704193401	-6.147011324

Trans-**2**, ground state

C	0.841206288	3.518028792	1.514504409
C	0.454598356	3.460965628	0.162238900
C	0.792298217	2.311045716	2.241041317
C	0.044219200	2.273801608	-0.396569746
C	0.374567495	1.153888160	1.632148917
H	0.471301149	4.351606718	-0.452269020
H	1.077812056	2.269820554	3.283595866
H	-0.271305453	2.187070005	-1.426895254
C	1.254922400	4.785893190	2.082371020
H	1.244335603	5.628768905	1.398596365
C	1.638915108	4.960981817	3.365509727
H	1.649352727	4.103442860	4.034191244
C	2.057877726	6.212801366	3.982439279
C	2.472461030	6.189997191	5.323568777
C	2.061720784	7.439568542	3.300643563
C	2.887241018	7.358068317	5.960391499
C	2.469726713	8.620450557	3.929495371
C	2.888236098	8.563805351	5.274502226
H	2.466143598	5.248665791	5.864772898
H	1.737184163	7.486481012	2.265856685
H	3.210020976	7.324476719	6.995322109
N	0.004778299	1.141910182	0.333647634
H	3.210191889	9.466359674	5.782394394
C	2.433423752	9.859300847	3.162120031
H	2.064274593	9.752884432	2.144689975
C	2.795945183	11.084054187	3.601385693
H	3.162182939	11.212685480	4.615490808
C	2.728547414	12.296634814	2.809849953
C	2.346305193	12.330271946	1.453385973
C	3.062715978	13.526841704	3.406169385
C	2.301774509	13.523879924	0.777797160
C	2.998309904	14.694085402	2.681267531

H	2.089589552	11.426790822	0.917786568
H	3.379892606	13.568078672	4.440551337
H	2.016317398	13.597127575	-0.261869068
H	3.246642948	15.659958622	3.098588306
N	2.621727172	14.685180782	1.388124820
C	2.501467063	15.953996124	0.644248316
H	3.192034517	16.656041099	1.110020183
H	2.847770890	15.759642191	-0.370854248
C	1.076053953	16.469894318	0.662386817
H	0.739942260	16.668698452	1.681862969
H	0.392394372	15.755714821	0.198796473
H	1.030080471	17.402418380	0.097222005
H	0.309134553	0.208646371	2.153137195
C	-0.395417317	-0.127489009	-0.303453855
H	-0.876906942	-0.726266884	0.469159098
H	-1.143729851	0.122727966	-1.054880825
C	0.794742313	-0.843218339	-0.911587520
H	1.536569331	-1.096424609	-0.151664942
H	1.274150344	-0.230458861	-1.678028648
H	0.451391807	-1.768783518	-1.377239265

Trans-***2***, excited state

C	0.814585551	3.420835275	1.569355731
C	0.285265628	3.346054555	0.263074602
C	0.896325499	2.209380532	2.291719008
C	-0.128645946	2.139970271	-0.256257509
C	0.468094195	1.033635171	1.720498748
H	0.189894784	4.235665405	-0.350304429
H	1.290437952	2.165675078	3.299795568
H	-0.543015897	2.045616123	-1.253796845
C	1.232247970	4.700852201	2.082825082
H	1.130378765	5.535653218	1.395963362
C	1.732534200	4.931710596	3.352076655
H	1.819059743	4.090936712	4.036669489
C	2.151728067	6.180716283	3.873772385
C	2.626601703	6.185531233	5.218516387
C	2.114696863	7.403736537	3.129476831
C	3.060204559	7.367621015	5.773964212
C	2.539185839	8.603090735	3.676147734
C	3.034447387	8.545293111	5.014352657
H	2.650716654	5.260015309	5.785207460
H	1.746453343	7.401824159	2.108945063
H	3.442645074	7.394792717	6.788762807
N	-0.038733754	1.004118296	0.468083752
H	3.430877593	9.450258043	5.463775854
C	2.508206332	9.846222251	2.910023844
H	2.382985283	9.732058196	1.837260746
C	2.586980307	11.090594279	3.463663411
H	2.639054751	11.173990275	4.548552272
C	2.588515145	12.351892931	2.783366957
C	2.586969042	12.495154272	1.364928030
C	2.610729295	13.564008910	3.522918702

C	2.602998162	13.730377382	0.786088863
C	2.625159639	14.778848371	2.899327040
H	2.589374902	11.629991892	0.712598957
H	2.619462798	13.544083230	4.607819501
H	2.607210226	13.869576771	-0.288696015
H	2.643486808	15.716402973	3.441531629
N	2.627419718	14.874759581	1.532665894
C	2.557059419	16.187377437	0.884337422
H	3.117439033	16.889944457	1.507495049
H	3.088243260	16.111493848	-0.068596455
C	1.125472508	16.657461170	0.680274305
H	0.597194452	16.743762110	1.634081366
H	0.568910473	15.967531642	0.039435436
H	1.123993035	17.640418387	0.202137415
H	0.513143479	0.084663663	2.243045067
C	-0.443366224	-0.290890747	-0.135324343
H	-0.813085134	-0.917728477	0.679302802
H	-1.285371856	-0.081237110	-0.799037038
C	0.710817281	-0.946604525	-0.874751397
H	1.548703617	-1.160922678	-0.205715923
H	1.067219276	-0.323595119	-1.699651068
H	0.364966325	-1.894476903	-1.294675357

Cis-***2***, ground state

C	0.942082119	3.647395898	1.573637661
C	0.728282947	3.740437514	0.185284636
C	1.023098364	2.350502158	2.119172026
C	0.603985820	2.605458398	-0.580636965
C	0.889689638	1.251674305	1.306891862
H	0.657941976	4.708087283	-0.294263195
H	1.185678449	2.188665578	3.176403447
H	0.435584534	2.631288433	-1.648157381
C	1.056370165	4.865592310	2.350500394
H	0.993355060	5.784573628	1.775060642
C	1.221114649	4.914341280	3.690238628
H	1.284116969	3.983903838	4.250006232
C	1.319164956	6.119685765	4.504333078
C	1.529995937	5.980930907	5.885990005
C	1.191950752	7.412002289	3.971616655
C	1.609453324	7.101316843	6.708266919
C	1.304262306	8.545447364	4.781778127
C	1.496974631	8.375933604	6.165110587
H	1.616126150	4.985478180	6.310484773
H	1.006388423	7.545639520	2.910615853
H	1.745760414	6.980413289	7.777405803
N	0.681741317	1.382356579	-0.020476919
H	1.527733656	9.245391691	6.813857926
C	1.128888230	9.864128263	4.160902421
H	0.382728106	9.884993891	3.369047550
C	1.720823713	11.048313083	4.424171855
H	1.331058102	11.908920200	3.887231435
C	2.841480978	11.372160651	5.299482555

C	3.838635939	10.460786063	5.694145727
C	3.000838407	12.706805992	5.714160119
C	4.891012828	10.882383701	6.471546044
C	4.071027141	13.072806144	6.497226321
H	3.803898369	9.423847311	5.389632162
H	2.282176165	13.463590156	5.426345415
H	5.676831318	10.217482781	6.800326384
H	4.226189922	14.085074616	6.843149303
N	4.994650521	12.166590603	6.870325613
C	6.172142317	12.600649230	7.648033731
H	5.840762924	13.420592898	8.284980030
H	6.453372204	11.765711538	8.289196672
C	7.305227583	13.021029130	6.734555903
H	7.004597840	13.843146964	6.082078591
H	7.644385863	12.187870128	6.115923408
H	8.146440527	13.358665945	7.342626086
H	0.937983610	0.238457885	1.679455540
C	0.611157180	0.178863078	-0.870955715
H	0.182434109	-0.613458458	-0.257693494
H	-0.089090389	0.399177709	-1.676497728
C	1.980352588	-0.197099935	-1.402275632
H	2.673536713	-0.413576799	-0.587052745
H	2.398824473	0.604685914	-2.013896496
H	1.890101373	-1.090232107	-2.023139952

Cis-***2***, excited state

C	0.924405476	3.566527749	1.516815199
C	0.645796349	3.619571142	0.135631383
C	0.994115801	2.284641086	2.102365679
C	0.450599710	2.461358146	-0.584091792
C	0.790015777	1.161745390	1.333501473
H	0.574383682	4.570330309	-0.381888449
H	1.199856447	2.143524323	3.156622586
H	0.230912891	2.464389588	-1.645960361
C	1.110335461	4.804641528	2.238039402
H	1.016221327	5.706663256	1.640530508
C	1.379676283	4.919173277	3.583089102
H	1.470939809	4.012608609	4.177370726
C	1.545388459	6.137702237	4.302465918
C	1.777609004	6.026200710	5.709964689
C	1.482006911	7.424577130	3.702437079
C	1.901335977	7.164500971	6.473878040
C	1.619661596	8.583211216	4.450137968
C	1.806962191	8.421505568	5.863763683
H	1.835088704	5.042457566	6.166175123
H	1.310198557	7.520609040	2.635502255
H	2.043921802	7.097088391	7.547138991
N	0.519671811	1.253772813	0.013138566
H	1.844180452	9.311640642	6.482451103
C	1.449200854	9.898832491	3.842213382
H	0.790218760	9.911492938	2.978409767
C	1.944168382	11.107335510	4.250243653

H	1.512271377	11.969613330	3.742887251
C	2.970349395	11.438624365	5.198472069
C	4.006783162	10.546207454	5.617745224
C	3.029105941	12.746953773	5.752483922
C	4.939184978	10.937606948	6.536927313
C	3.978025505	13.090946698	6.670180892
H	4.124539837	9.572029165	5.157885166
H	2.300377667	13.495885415	5.459739821
H	5.750251900	10.291709502	6.853742890
H	4.038621010	14.077812716	7.112285877
N	4.923025720	12.182921800	7.092005759
C	5.989816362	12.610467363	8.001591585
H	5.543439960	13.291800587	8.731727743
H	6.324776014	11.726919504	8.551854960
C	7.147368983	13.273008600	7.269912085
H	6.816050324	14.161952507	6.725836543
H	7.609853312	12.584937715	6.556468477
H	7.910593324	13.581387503	7.988991223
H	0.831655203	0.160170967	1.746734505
C	0.351823069	0.018300316	-0.794816974
H	-0.097042603	-0.730124284	-0.137872153
H	-0.374828750	0.249215946	-1.577171842
C	1.674740598	-0.455280841	-1.372135440
H	2.394940711	-0.697459302	-0.585786305
H	2.115613176	0.291899107	-2.037678569
H	1.497532850	-1.362060449	-1.955899833

DHP-***2***, ground state

C	0.712358135	3.412787940	1.539754290
C	-0.301011569	3.447247610	0.562575784
C	1.350611539	2.173342814	1.751162181
C	-0.629749828	2.313414500	-0.141733837
C	0.980009069	1.072438427	1.020272974
H	-0.836743239	4.365678566	0.358865141
H	2.131987420	2.061006101	2.490814056
H	-1.399432414	2.295092880	-0.900529165
C	1.032920415	4.620362619	2.268683840
H	0.432790684	5.489966841	2.016842692
C	1.995826831	4.717390170	3.217522435
H	2.567056459	3.829475329	3.478770643
C	2.341366832	5.911037554	3.962072049
C	3.295094951	5.779923979	5.059150969
C	1.880355193	7.164365473	3.628087069
C	3.594238159	6.815668186	5.858062320
C	2.243121272	8.320464619	4.372086192
C	2.885115338	8.132020313	5.729599522
H	3.779063126	4.818216190	5.201009113
H	1.281450621	7.308184163	2.733506752
H	4.320969391	6.696559818	6.656026547
N	0.004105881	1.146670206	0.089602228
H	2.038736884	8.112090975	6.449945226
C	1.938293330	9.584282769	3.926614111

H	1.373143099	9.700302348	3.007203005
C	2.332642201	10.742007209	4.651505428
H	2.003288127	11.719074028	4.311632987
C	3.162016952	10.655829396	5.735284839
C	3.766248737	9.319906385	6.114400981
C	3.609806062	11.791312517	6.472219308
C	4.098690166	9.286415030	7.563600830
C	4.267518700	11.642644223	7.645290502
H	4.737324763	9.228413457	5.580177744
H	3.384263252	12.791654801	6.121360103
H	4.140669926	8.352126720	8.110638371
H	4.620291984	12.457572860	8.258710825
N	4.393125074	10.363788938	8.217656970
C	4.835043391	10.303141129	9.626092817
H	4.201778171	10.995587755	10.182756870
H	4.629862797	9.292277819	9.975224116
C	6.301743133	10.663665788	9.750122943
H	6.499768771	11.673561639	9.384574225
H	6.930619929	9.960577319	9.201153802
H	6.583133507	10.628824084	10.803929223
H	1.438839147	0.102484512	1.151222409
C	-0.326627319	-0.041378760	-0.719962284
H	-0.183890131	-0.909529729	-0.076941545
H	-1.386695807	0.028678013	-0.962583916
C	0.534138892	-0.114756491	-1.965967810
H	1.593498351	-0.183788912	-1.710778404
H	0.381422893	0.758971311	-2.602478857
H	0.260560856	-1.005129109	-2.534766082

DHP-***2***, excited state (near-CI structure)

C	0.812778574	3.491466674	1.467601488
C	0.181273124	3.558901584	0.205970760
C	1.145522581	2.195383812	1.922790887
C	-0.093332923	2.419543179	-0.510531666
C	0.847174132	1.090599944	1.165041985
H	-0.107971130	4.515052054	-0.216438848
H	1.640231593	2.031292787	2.872382359
H	-0.582838854	2.445964960	-1.477491011
C	1.069628046	4.708839372	2.193966164
H	0.791719875	5.622602130	1.674893677
C	1.594974768	4.781742479	3.447825539
H	1.844061197	3.849978869	3.953038003
C	1.847137593	5.964838909	4.222642913
C	2.335001633	5.820221719	5.559082417
C	1.624605678	7.271101879	3.755045654
C	2.599024302	6.885725826	6.373016477
C	1.855197311	8.387344810	4.550451150
C	2.359300545	8.250296602	5.942257206
H	2.488303109	4.820377031	5.957920489
H	1.290933088	7.424580277	2.732591014
H	2.931342843	6.690876001	7.388754269
N	0.231313107	1.198316158	-0.034240022

H	1.708291841	8.808118485	6.630403343
C	1.688440878	9.707270591	4.063297633
H	1.155444988	9.843795653	3.125426150
C	2.167720335	10.807837975	4.763785890
H	1.844838302	11.812078485	4.504229191
C	3.112875769	10.660799496	5.791076119
C	3.707071441	9.306402634	6.007814245
C	3.527000479	11.715213226	6.632674711
C	4.557640483	9.188087811	7.160487737
C	4.285071114	11.459650355	7.740949220
H	4.225975881	8.968057747	5.102179283
H	3.175114650	12.727897193	6.467937723
H	5.085013460	8.266081589	7.378881515
H	4.536976607	12.228781793	8.460890485
N	4.749782993	10.191916221	8.024881569
C	5.592723860	10.009736580	9.226666521
H	5.065378598	10.476356754	10.063682892
H	5.637418834	8.937577786	9.428817207
C	6.983715985	10.592589827	9.047284054
H	6.956149259	11.670310424	8.863844774
H	7.512215929	10.114314000	8.218177564
H	7.559824834	10.427414853	9.961081547
H	1.088828275	0.085044670	1.490140718
C	-0.025445293	-0.010618448	-0.848023587
H	-0.242963566	-0.824198338	-0.152244045
H	-0.932603653	0.180171216	-1.425736680
C	1.151149758	-0.344574895	-1.748235826
H	2.055850839	-0.547445584	-1.168528717
H	1.360725062	0.465030852	-2.452687164
H	0.914613065	-1.241044272	-2.326761763

Phen-***2***, ground state

C	0.774223528	3.437727582	1.487876025
C	-0.163496093	3.506075862	0.442175416
C	1.307289770	2.169182460	1.787025303
C	-0.530647304	2.369244255	-0.239280208
C	0.905953611	1.067461598	1.072910813
H	-0.613274126	4.451665546	0.168416834
H	2.030479643	2.035909196	2.579875598
H	-1.254074666	2.372301838	-1.042283022
C	1.129562687	4.650595705	2.200680542
H	0.659401755	5.558405051	1.834472551
C	1.962639600	4.701001218	3.261236524
H	2.401001024	3.779828632	3.637513908
C	2.331469908	5.900857245	4.003120709
C	3.040872005	5.748614259	5.218802861
C	2.019208657	7.187910645	3.575980125
C	3.398091161	6.838037900	5.982975598
C	2.379569172	8.313367128	4.335854316
C	3.073988381	8.143924204	5.565929199
H	3.301005256	4.750237866	5.555373015
H	1.495448274	7.346791894	2.638901916

H	3.932421256	6.675795010	6.911849169
N	-0.001059534	1.172418892	0.079023201
C	2.050816969	9.631171706	3.877239248
H	1.525292987	9.723594964	2.931983078
C	2.375282274	10.743285190	4.591841811
H	2.113742853	11.732612758	4.234385701
C	3.059038767	10.618106114	5.836548446
C	3.410705574	9.328657864	6.326064492
C	3.395250423	11.745623969	6.615916178
C	4.077435802	9.256737844	7.550813451
C	4.045082896	11.590696703	7.807544643
H	3.136446435	12.741346032	6.279878594
H	4.380922491	8.318588068	7.992957042
H	4.326911742	12.414638858	8.447568491
N	4.377900817	10.349219575	8.253024961
C	5.110960385	10.232126443	9.531496192
H	4.533643645	10.781480323	10.276592147
H	5.097877102	9.177375653	9.801025473
C	6.524700995	10.763074792	9.410605804
H	6.532396261	11.816905759	9.123094030
H	7.102886575	10.193962037	8.680281444
H	7.016359525	10.677830009	10.381274008
H	1.282930102	0.073546583	1.266832769
C	-0.369900995	-0.024913508	-0.701130068
H	-0.316679461	-0.872330552	-0.018342987
H	-1.409218554	0.102718333	-1.002221053
C	0.545210644	-0.204954995	-1.896083111
H	1.584648749	-0.325273524	-1.584925069
H	0.477740388	0.645830610	-2.576601991
H	0.245958023	-1.102445061	-2.439997555

*Trans-to-cis-**2*** TS, excited state

C	0.823486152	3.359682943	1.387265470
C	0.564121668	3.224462926	0.008425279
C	0.827675132	2.166902590	2.141624152
C	0.346699162	1.986623844	-0.550042340
C	0.603748581	0.956422328	1.532847983
H	0.536134123	4.095644782	-0.637194901
H	0.987924682	2.171469544	3.213090353
H	0.150277791	1.850702756	-1.607684732
C	1.058430266	4.676807031	1.943803137
H	0.906946730	5.504864877	1.255496335
C	1.485857255	4.925024871	3.203457857
H	1.703039892	4.085220956	3.860986455
C	1.743541809	6.231377245	3.792958404
C	2.478119729	6.264651889	5.002878833
C	1.315880589	7.435215885	3.236774717
C	2.810768027	7.470526915	5.618556817
C	1.648575742	8.676391462	3.839746308
C	2.417039399	8.662804223	5.046013358
H	2.805215402	5.329285139	5.449382777
H	0.713537497	7.443401554	2.332842633

H	3.383526109	7.468142863	6.540176387
N	0.366303108	0.867386102	0.204572472
H	2.680952111	9.610838304	5.508030803
C	1.225329994	9.897592568	3.276030385
H	0.590307907	9.841905282	2.386173738
C	1.600913205	11.194705552	3.755799367
H	2.666963371	11.429345871	3.823709902
C	0.705939499	12.235695992	4.061319543
C	-0.712852881	12.069488869	4.034845018
C	1.175556562	13.544536790	4.384850110
C	-1.540599480	13.124671437	4.304510247
C	0.295115730	14.558096701	4.638676736
H	-1.148513610	11.103439004	3.800755685
H	2.239562030	13.753974894	4.421923681
H	-2.621053260	13.031692689	4.298386725
H	0.619409172	15.563190817	4.884080607
N	-1.051861482	14.359113902	4.592200566
C	-1.970603866	15.463033652	4.922339391
H	-1.510949856	16.386225564	4.559810787
H	-2.885056972	15.307463774	4.344144254
C	-2.261572885	15.536038261	6.413575398
H	-1.348478131	15.705954006	6.990923545
H	-2.736428515	14.618795365	6.773029636
H	-2.943185373	16.368498596	6.607046845
H	0.598093429	0.022038981	2.082980751
C	0.185224633	-0.461213537	-0.425915232
H	-0.357126843	-1.082958896	0.290588150
H	-0.465293637	-0.316600800	-1.291473018
C	1.514178139	-1.083075063	-0.818533590
H	2.158739203	-1.236422580	0.051454348
H	2.047405251	-0.464061347	-1.545284577
H	1.330143280	-2.058180755	-1.276485563

*Cis-to-trans-**2*** TS, excited state

C	0.803009047	3.438052762	1.537389548
C	-0.110084802	3.333497863	0.469229788
C	1.436830302	2.243064484	1.936438716
C	-0.365176935	2.118964522	-0.123885067
C	1.144573855	1.056229464	1.308838833
H	-0.637284981	4.208360574	0.104247583
H	2.166198214	2.224115977	2.737380921
H	-1.067907309	2.005932198	-0.941937184
C	1.029821545	4.731361594	2.153064350
H	0.519069689	5.563909290	1.675322081
C	1.769608852	4.949499699	3.263679168
H	2.227544431	4.099685568	3.766908612
C	2.000049565	6.230142086	3.919136670
C	2.547562106	6.217960618	5.228791205
C	1.712790989	7.456888616	3.331354713
C	2.753177117	7.395908685	5.938241577
C	1.937089707	8.677955738	4.023657444
C	2.451648247	8.619132352	5.363012423

H	2.790029326	5.265123646	5.691817532
H	1.330201360	7.508199033	2.315869171
H	3.139545668	7.353036374	6.951618321
N	0.251470890	0.994771917	0.296347817
H	2.559281087	9.537236103	5.930629687
C	1.655616082	9.904493433	3.395032697
H	1.195233097	9.863939704	2.404506836
C	1.838444544	11.198572593	3.982307205
H	0.971948222	11.863273199	4.008930216
C	3.046421175	11.692892260	4.490728400
C	4.271386426	10.945965390	4.432216398
C	3.116158092	12.958676989	5.150540119
C	5.409320843	11.443066026	5.015056577
C	4.283453233	13.391113734	5.708106684
H	4.324695131	10.006032772	3.895994912
H	2.234080893	13.585897728	5.226764125
H	6.354550619	10.912039233	4.981197554
H	4.369721018	14.343742612	6.218129801
N	5.422225434	12.634953795	5.655189300
C	6.675409786	13.169023245	6.217895345
H	6.418212588	13.688706271	7.145059403
H	7.299114463	12.313226585	6.487664028
C	7.391233974	14.092528810	5.244152284
H	6.773202346	14.955587692	4.981284981
H	7.664965257	13.566779105	4.325161789
H	8.308014604	14.464573311	5.708864462
H	1.611117827	0.119504560	1.592683572
C	-0.004034205	-0.293897516	-0.390387405
H	0.091855548	-1.079763116	0.362442804
H	-1.046815769	-0.278844385	-0.715841464
C	0.946514167	-0.509692288	-1.554954400
H	1.988417407	-0.536497781	-1.224020877
H	0.838573042	0.272222682	-2.311780458
H	0.718956260	-1.468613219	-2.027278093

*Trans-to-cis-**2*** near-CI region

C	0.823472434	3.345115761	1.380290299
C	0.550986777	3.219102298	0.005019850
C	0.840840077	2.156611128	2.136574906
C	0.330420810	1.979543184	-0.557318241
C	0.614153444	0.942832440	1.529517145
H	0.513484183	4.092555999	-0.637650625
H	1.012565899	2.161297696	3.207208971
H	0.123006008	1.847370391	-1.613560966
C	1.061115958	4.671107670	1.940400931
H	0.895844604	5.496646113	1.249710830
C	1.501270801	4.920570101	3.196495230
H	1.730284798	4.082977296	3.855765036
C	1.756798860	6.235209795	3.779556904
C	2.508673290	6.290128078	4.988351959
C	1.317150666	7.443800577	3.239189996
C	2.848305436	7.472633621	5.618779900

C	1.657842569	8.667813211	3.858567067
C	2.438009135	8.674526964	5.056612527
H	2.846229371	5.353579102	5.432613956
H	0.704077477	7.470727317	2.344124933
H	3.429947337	7.452566705	6.534626065
N	0.362168790	0.856849159	0.199983151
H	2.680669729	9.638162222	5.495597617
C	1.223082820	9.910807277	3.307472785
H	0.572658653	9.825041245	2.414817873
C	1.588638251	11.148609524	3.766153407
H	2.439884500	11.577109653	3.247717710
C	0.898670342	11.904075342	4.717548207
C	-0.249632966	11.417806410	5.449521609
C	1.258162084	13.269349887	5.039656763
C	-0.909277926	12.203102412	6.342098703
C	0.552208020	13.995617121	5.941478065
H	-0.612417062	10.402059443	5.296261380
H	2.103486025	13.737636555	4.545548578
H	-1.771178187	11.841011433	6.895691174
H	0.814476866	15.021408498	6.184011637
N	-0.543903698	13.490344292	6.602908401
C	-1.247198026	14.290405026	7.607669542
H	-1.218552959	15.330533006	7.268219401
H	-2.295021326	13.975392509	7.595215988
C	-0.647846971	14.150723799	8.996545335
H	0.395264055	14.480507356	9.013144046
H	-0.691754829	13.114252171	9.344664074
H	-1.206158952	14.768033373	9.706549338
H	0.617630482	0.008675490	2.080392949
C	0.177424680	-0.474903931	-0.431176067
H	-0.355223674	-1.098794788	0.290763353
H	-0.483081842	-0.331148384	-1.289244437
C	1.505411607	-1.089658190	-0.838221937
H	2.160028388	-1.241997316	0.024377711
H	2.028499518	-0.468200908	-1.570306945
H	1.319055098	-2.064802530	-1.295211121
